# The home electronic media environment and parental safety concerns: relationships with outdoor time after school and over the weekend among 9–11 year old children

**DOI:** 10.1186/s12889-018-5382-0

**Published:** 2018-04-05

**Authors:** Hannah J. Wilkie, Martyn Standage, Fiona B. Gillison, Sean P. Cumming, Peter T. Katzmarzyk

**Affiliations:** 1 Centre for Motivation and Health Behaviour Change, Department for Health, University of Bath, Bath, UK; 2 Pennington Biomedical Research Center, Baton Rouge, USA

**Keywords:** Outdoor time, Physical activity, Children, Screen time, Safety concerns

## Abstract

**Background:**

Time spent outdoors is associated with higher physical activity levels among children, yet it may be threatened by parental safety concerns and the attraction of indoor sedentary pursuits. The purpose of this study was to explore the relationships between these factors and outdoor time during children’s discretionary periods (i.e., after school and over the weekend).

**Methods:**

Data from 462 children aged 9–11 years old were analysed using generalised linear mixed models. The odds of spending > 1 h outdoors after school, and > 2 h outdoors on a weekend were computed, according to demographic variables, screen-based behaviours, media access, and parental safety concerns. Interactions with sex and socioeconomic status (SES) were explored.

**Results:**

Boys, low SES participants, and children who played on their computer for < 2 h on a school day had higher odds of spending > 1 h outside after school than girls, high SES children and those playing on a computer for ≥2 h, respectively. Counterintuitive results were found for access to media devices and crime-related safety concerns as both of these were positively associated with time spent outdoors after school. A significant interaction for traffic-related concerns*sex was found; higher road safety concerns were associated with lower odds of outdoor time after school in boys only. Age was associated with weekend outdoor time, which interacted with sex and SES; older children were more likely to spend > 2 h outside on weekends but this was only significant among girls and high SES participants.

**Conclusions:**

Our results suggest that specific groups of children are less likely to spend their free time outside, and it would seem that only prolonged recreational computer use has a negative association with children’s outdoor time after school. Further research is needed to explore potential underlying mechanisms, and parental safety concerns in more detail.

## Background

Time spent outdoors has consistently been associated with higher physical activity levels, [1–7] and in a recent position statement, put forward by Tremblay and colleagues, [8] active outdoor play in the natural environment was recognised as a fundamental component of children’s health and development. Not only is there more space for children to be physically active outdoors, [9] but access to sedentary pursuits is also minimised because activities such as TV viewing and playing computer games are usually performed indoors. [7] It is, therefore, not surprising that children engage in significantly more physical activity outside the home rather than when they are indoors [9–11].

Despite the benefits of outdoor play, research suggests that children have less freedom to play outside than they did in previous generations [8, 12, 13]. Many parents and grandparents cite that children no longer play traditional games or know how to ride a bike, both of which were frequent pastimes in their own childhood [14]. According to qualitative data, [15, 16] previously reported barriers to outdoor play have included safety concerns, a lack of time, and greater pressure on academic study, as well as access to digital entertainment in the home. However, there is little support in the literature for an association between safety perceptions and children’s physical activity, [5, 12, 17, 18] and a negative, but weak, relationship was observed between screen-based behaviours and physical activity in a meta-analysis [19]. The authors concluded that the effect is therefore unlikely to be clinically relevant [19].

A key criticism of past work is that general measures of overall physical activity tend to be assessed, [4, 17] as opposed to certain types of physical activity performed during specific times [20]. In terms of the extant literature on time spent outdoors specifically, which is positively associated with physical activity, [2, 3, 21] overall measures of this behaviour have also been explored (e.g., [22–24]). Though it may be that screen-based pursuits are more likely to compete for children’s time outdoors after school or on weekends, when access to media-based entertainment is likely to be more prominent [4]. This notion is supported by two Australian studies; one found that the majority (78%) of time after school was spent indoors among 5–7 year olds, [9] while the other reported that this period contributed to 84% of children’s daily screen time among a larger sample of 8–9 year olds. [25] Furthermore, different aspects of parental perceptions of safety, such as traffic- and crime-related concerns, are rarely considered separately. [18] Thus, important relationships may have been missed in previous research because associations may differ between the two.

Consequently, the purpose of this study was to explore the relationships between indoor sedentary pursuits (specifically TV viewing, recreational computer use, and access to home electronic media devices), and parental perceived crime- and traffic-related safety concerns, with outdoor time after school and over the weekend. Given potential differences between boys and girls and socioeconomic groups, highlighted in past research, [6, 26] we also tested for interactions with sex and socioeconomic status (SES).

## Methods

### Participants

Children in Years 5 and 6 (age 9–11 years) at schools across Bath and North East Somerset and West Wiltshire were recruited as part of the International Study of Childhood Obesity, Lifestyle and the Environment (ISCOLE) [27]. Parental consent and child assent were obtained from all participants prior to data collection, which took place from September 2011 to January 2013 during term time. Ethical approval was granted from the University of Bath Research Ethics Approval Committee for Health (REACH).

### Measures

#### Outdoor time

Participants completed a Diet and Lifestyle Questionnaire, [27] whereby they were asked how much time they spend outside ‘on a school day after school before bedtime’, and ‘on a weekend day’. Six response options were available: ‘< 1 h’; ‘1 h’; ‘2 h’; ‘3 h’; ‘4 h’; ‘5 or more h’. A large amount of variation exists in the literature with regards to how time spent outdoors is expressed. In many studies, this behaviour has been dichotomised but different criteria have been applied to govern ‘low’ and ‘high’ amounts of time spent outdoors (e.g., ‘low’ amounts of time outdoors have been classified as < 0.5 h/day [28]; < 1 h/day [6]; < 2 h/day [29, 30] and ≤ 2 h/day [24]). As such, we decided to conduct a frequency analysis, akin to Stone and Faulkner, [6] and chose the following categories for outdoor time after school: ≤ 1 h/day versus > 1 h/day, and for weekend outdoor time: ≤ 2 h/day versus > 2 h/day. Although different categories were chosen for after school and weekend outdoor time, these criteria were deemed as suitable given that there is more free time available over the weekend, and we wanted to capture differences for those spending several hours outdoors. A similar approach has been applied previously by Cleland et al., [31] who used different criteria for different seasons, as more time was spent outside during warmer months than during cooler months.

#### Screen time

Participants also responded to four questions from the Youth Risk Behavior Surveillance System regarding the time spent watching TV and playing on a computer on school and weekend days specifically [32]. This scale was deemed to possess adequate reliability and validity according to a study on 11–15 year olds [33]. Available options included: ‘I did not watch TV/play video or computer games or use a computer other than for school work on school/weekend days’; ‘< 1 h’; ‘1 h’; ‘2 h’; ‘3 h’; ‘4 h’; and ‘5 or more h’. Children were categorised into high, medium, and low screen time groups based on the following criteria: school day TV viewing: < 2, 2, and ≥ 3 h/day; weekend TV viewing: < 2, 2–3, and ≥ 4 h/day; school and weekend recreational computer use: None, < 2, and ≥ 2 h/day. These categories were chosen in line with screen time recommendations, [34] previous research, [35, 36] and based on a frequency analysis of the current data.

#### Home electronic media environment

A questionnaire was also administered to the child’s parent(s)/guardian(s), [27] which included six items from the Neighborhood Impact on Kids study survey, [37] regarding their child’s access to specific electronic devices. Three related to whether their child had the following items in his/her bedroom: 1) a computer; 2) a TV; and 3) a video game system (non-handheld; PlayStation, Xbox etc.). The remaining three items asked if their child had use of the following devices, not restricted to their bedroom: 1) a mobile phone or 2-way radio (walkie-talkie); 2) music systems (iPod, stereo, radio, etc.); and 3) handheld videogame players (Game Boy, DS etc.). Parents responded either ‘yes’ or ‘no’ to each item. An overall ‘media access’ score was computed by summing the total number of devices that each child had access to. Participants were then split into one of three media access categories using the following criteria: low (access to 1 or no devices), average (access to 2–4 devices), and high (access to 5 or 6 devices). These categories were chosen based on the premise that 8–11 year olds, across the United Kingdom (UK), own an average of 3 devices [38].

#### Parental safety concerns

Data were also obtained from parents/guardians on their perceptions of safety concerns within the area where they live. Items were adapted from the Neighbourhood Environment Walkability Scale for Youth, [39] which consists of 5 items assessing crime-related safety concerns (e.g., ‘I’m afraid of my child being taken or hurt by a stranger on local streets’) and 5 items pertaining to traffic-related safety concerns (e.g., ‘Most drivers go faster than the posted speed limits’). Each item included a 4-point Likert Scale (0–3) ranging from ‘Strongly Disagree’ to ‘Strongly Agree’. The mean of available items was computed for those with responses to at least 4 of the items in each subscale; higher scores represent greater concerns.

#### Demographic variables

Parents/guardians were also asked to provide information on their highest educational attainment in addition to their child’s date of birth and gender. Age at the time of data collection was calculated from their date of birth. The highest parental education level was used as an indicator of SES; participants were classified as having either a high (A Levels or University Degree) or low (General Certificate for Secondary Education (GCSEs) or less) SES.

### Statistical analysis

SAS Studio 3.5 (SAS Institute Inc., Cary, NC, USA, 2012–2016) was used for all analyses. Participants with missing data for any variables were not included in the analytic sample. Descriptive statistics were computed for the total sample and by sex, and compared between those included and excluded using an independent samples t-test for continuous variables and chi-squared tests for categorical variables. Generalised linear mixed models were employed for the main analysis using the GLIMMIX procedure, and results are presented as Odds Ratios (OR). Schools were treated as random effects in all models given the study design to adjust for potential clustering at the school level (ICCs: 0.09 and 0.03 for after school and weekend outdoor time, respectively). Simple associations were tested first, exploring the relationship between each independent variable and outcome variables, adjusting for covariates (age, sex, and SES) only. All variables were then entered into a mutually adjusted model; checks for multicollinearity were performed and no problems were identified. Finally, interactions with sex and SES were explored, as were relationships between specific media devices and each outcome variable.

## Results

Consent was obtained from 541 participants but following eight withdrawals, and exclusion of those with invalid data, the analytic sample was comprised of 462 participants with complete data. No significant differences were found in terms of age, sex, outdoor time after school or outdoor time on weekends between those included and excluded from the analysis. Descriptive statistics are displayed in Table 1. The average age of participants was 10.9 (± 0.5) years and a higher proportion of children had parents with a high, versus low education level (71% vs. 29%). Over half (52.2%) of the analytic sample reported spending time outdoors for > 1 h after school and 61.9% spent > 2 h outside on a weekend.

**Table 1:** Descriptive characteristics of the analytic sample: Mean (SD) or %

	Total Sample (*N* = 462)	Boys (*N* = 208)	Girls (*N* = 254)
Age	10.9 (0.5)	10.9 (0.4)	10.9 (0.5)
SES (Highest parental education)			
Low (GCSEs or less)	29.0	24.5	32.7
High (A Levels/University Degree)	71.0	75.5	67.3
Outdoor Time (all categories)			
After school			
0 h/day	21.4	16.4	25.6
1 h/day	26.4	28.4	24.8
2 h/day	26.4	28.4	24.8
3 h/day	14.3	14.4	14.2
4 h/day	7.4	8.7	6.3
5 h/day	4.1	3,9	4.3
Weekend			
0 h/day	4.1	2.9	5.1
1 h/day	11.3	8.7	13.4
2 h/day	22.7	24.0	21.7
3 h/day	22.1	21.2	22.8
4 h/day	20.4	23.1	18.1
5 h/day	19.5	20.2	18.9
Outdoor Time (using cut-offs)			
After school (> 1 h/d)	52.2	55.3	49.6
Weekend (> 2 h/d)	61.9	64.4	59.8
TV Viewing: School day / Weekend			
Low	54.1 / 28.6	54.3 / 33.2	53.9 / 24.8
Medium	30.7 / 54.6	28.4 / 48.1	32.7 / 59.8
High	15.2 / 16.9	17.3 / 18.8	13.4 / 15.4
Computer use: School day / Weekend			
Low	22.9 / 13.6	16.4 / 10.1	28.4 / 16.5
Medium	50.9 / 44.2	45.2 / 32.7	55.5 / 53.5
High	26.2 / 42.2	38.5 / 57.2	16.1 / 29.9
Media access			
Low (1 or no electronic devices)	13.0	14.9	11.4
Average (2–4 electronic devices)	70.8	63.5	76.8
High (5 or 6 electronic devices)	16.2	21.6	11.8
Crime-related safety concerns score	1.1 (0.6)	1.0 (0.6)	1.2 (0.7)
Traffic-related safety concerns score	1.3 (0.5)	1.4 (0.5)	1.3 (0.5)

*GCSEs* General Certificate for Secondary Education, *SES* socioeconomic status, *TV* television

School day TV viewing categories: Low = < 2 h/d; Medium = 2 h/d; High = ≥ 3 h/d. Weekend TV viewing categories: Low = < 2 h/d; Medium = 2–3 h/d; High = ≥ 4 h/d. School and Weekend Computer categories: Low = None; Medium = < 2 h/d; High = ≥ 2 h/d

### Outdoor time after school

SES, media access, and crime-related safety concerns were associated with outdoor time after school in the simple models (Table 2). These variables remained significant in the mutually adjusted model. In comparison to high SES participants, low SES children were 1.77 times more likely to spend > 1 h outside after school, and children with access to a low number of electronic devices (0 or 1) were less likely to report a high level of time outdoors after school than those with high access to several electronic devices (5 or 6). No significant difference was found between the average and high media access groups. A one unit increase in the crime-related safety concerns score was associated with 1.51 higher odds of spending > 1 h outside after school. Sex and time spent on a computer on a school day were significantly associated with time outdoors after school in the mutually adjusted model. Compared to girls, boys were 1.72 times more likely to spend a high level of time outdoors after school, and children who spent < 2 h of their time playing on a computer on school days were 1.98 times more likely to spend > 1 h outside after school than those reporting 2 or more hours of computer use. Although the overall effect of TV viewing on a school day was not statistically significant, those watching TV for < 2 h/school day displayed lower odds of time outdoors after school than those watching TV for 3 or more hours.

**Table 2:** Odds associated with spending > 1 h/day outdoors after school (*N* = 462): ORs (95% CIs)

	Model 1		Model 2	
	OR (95% CI)	*p*	OR (95% CI)	*p*
Demographics				
Age	0.89 (0.54–1.46)	0.641	0.84 (0.51–1.39)	0.499
Sex (Ref = Girls)	1.37 (0.93–2.03)	0.111	1.72 (1.12–2.65)^*^	*0.014*
SES (Ref = High education)	1.97 (1.26–3.08)^*^	*0.003*	1.77 (1.12–2.80)^*^	*0.015*
Home electronic media environment				
School day TV viewing (Ref = High; ≥ 3 h/d)	1	0.236	1	0.116
Mid (2 h/d)	0.70 (0.38–1.29)		0.58 (0.31–1.09)	
Low (< 2 h/d)	0.62 (0.35–1.08)		0.54 (0.30–0.97)^*^	
School day computer use (Ref = High; ≥ 2 h/d)	1	0.168	1	*0.030*
Mid (< 2 h/d)	1.57 (0.97–2.54)		1.98 (1.20–3.30)^*^	
Low (None)	1.24 (0.70–2.19)		1.72 (0.94–3.15)	
Media access (Ref = High; 5–6 devices)	1	*0.013*	1	*0.014*
Average (2–4 devices)	0.83 (0.47–1.44)		0.83 (0.47–1.46)	
Low (0–1 devices)	0.34 (0.15–0.75)^*^		0.33 (0.15–0.75)^*^	
Parental safety concerns				
Crime-related	1.47 (1.08–2.01)^*^	*0.015*	1.51 (1.09–2.10)^*^	*0.013*
Traffic-related^a^	0.96 (0.66–1.39)	0.812	0.82 (0.55–1.21)	0.312

*Ref* reference category, *SES* socioeconomic status, *TV* television

Model 1: Simple associations between each independent variable and outdoor time after school, adjusting for covariates (age, sex and SES). Odds ratios for demographic variables (age, sex and SES) are therefore taken from a model in which all three were included simultaneously. Schools were treated as random effects in all models

Model 2: Mutually adjusted model with all independent variables entered simultaneously, with schools treated as random effects

Effects of continuous variables are assessed as one unit offsets from the mean

^*^p < 0.05

^a^A significant interaction for traffic-related safety concerns*sex (p = 0.022) was found; see text for details

*P* values taken from Type 3 Tests of Fixed Effects; italic font indicates significant result

A significant interaction by sex was found for traffic-related safety concerns (*p* = 0.022). A unit increase in the traffic-related safety concerns score was associated with lower odds of spending more time outdoors after school in boys only (OR = 0.52, 0.28–0.97; *p* = 0.040). No significant relationship was observed for girls (OR = 1.36, 0.84–2.21; *p* = 0.207).

### Weekend outdoor time

Only age was significant in both the simple and mutually adjusted models for weekend outdoor time (Table 3). Older age was associated with higher odds of spending > 2 h outdoors on a weekend, though significant interactions by sex (*p* = 0.009) and SES (*p* = 0.027), showed that the relationship for age was only significant in girls (OR = 2.54, 1.43–4.51; *p* = 0.002) and high SES participants (OR = 2.25, 1.34–3.77; *p* = 0.002). Equivalent odds ratios for boys and low SES participants were 0.79 (0.39–1.62; *p* = 0.521) and 0.68 (0.27–1.71; *p* = 0.410), respectively.

**Table 3:** Odds associated with spending > 2 h/day outdoors on weekends (*N* = 462): ORs (95% CIs)

	Model 1		Model 2	
	OR (95% CI)	*p*	OR (95% CI)	*p*
Demographics				
Age^a^	1.59 (1.02–2.48)^*^	*0.039*	1.61 (1.01–2.57)^*^	*0.045*
Sex (Ref = Girls)	1.24 (0.84–1.83)	0.276	1.26 (0.82–1.92)	0.292
SES (Ref = High education)	1.22 (0.79–1.89)	0.359	1.19 (0.76–1.87)	0.454
Home electronic media environment				
Weekend TV viewing (Ref = High; ≥ 4 h/d)	1	0.184	1	0.203
Mid (2–3 h/d)	0.62 (0.35–1.08)		0.63 (0.36–1.11)	
Low (< 2 h/d)	0.59 (0.32–1.08)		0.58 (0.31–1.09)	
Weekend computer use (Ref = High; ≥ 2 h/d)	1	0.309	1	0.297
Mid (< 2 h/d)	0.80 (0.52–1.22)		0.86 (0.55–1.35)	
Low (None)	1.23 (0.65–2.30)		1.40 (0.72–2.71)	
Media access (Ref = High; 5–6 devices)	1	0.957	1	0.953
Mid (2–4 devices)	1.05 (0.60–1.81)		1.09 (0.62–1.90)	
Low (0–1 devices)	1.12 (0.53–2.38)		1.11 (0.51–2.39)	
Parental safety concerns				
Crime-related	1.16 (0.86–1.57)	0.334	1.14 (0.83–1.57)	0.416
Traffic-related	1.02 (0.70–1.49)	0.902	1.01 (0.69–1.49)	0.956

*Ref* reference category *SES* socioeconomic status, *TV* television

Model 1: Simple associations between each independent variable and weekend outdoor time, adjusting for covariates (age, sex and SES). Odds ratios for demographic variables (age, sex and SES) are therefore taken from a model in which all three were included simultaneously. Schools were treated as random effects in all models

Model 2: Mutually adjusted model with all independent variables entered simultaneously, with schools treated as random effects

Effects of continuous variables are assessed as one unit offsets from the mean

^*^p < 0.05

^a^A significant interaction for age*sex (*p* = 0.009) and for age*SES (*p* = 0.027) was found; see text for details

P values taken from Type 3 Tests of Fixed Effects; italic font indicates significant result

### Associations with access to specific electronic media devices

**Fig. 1:**
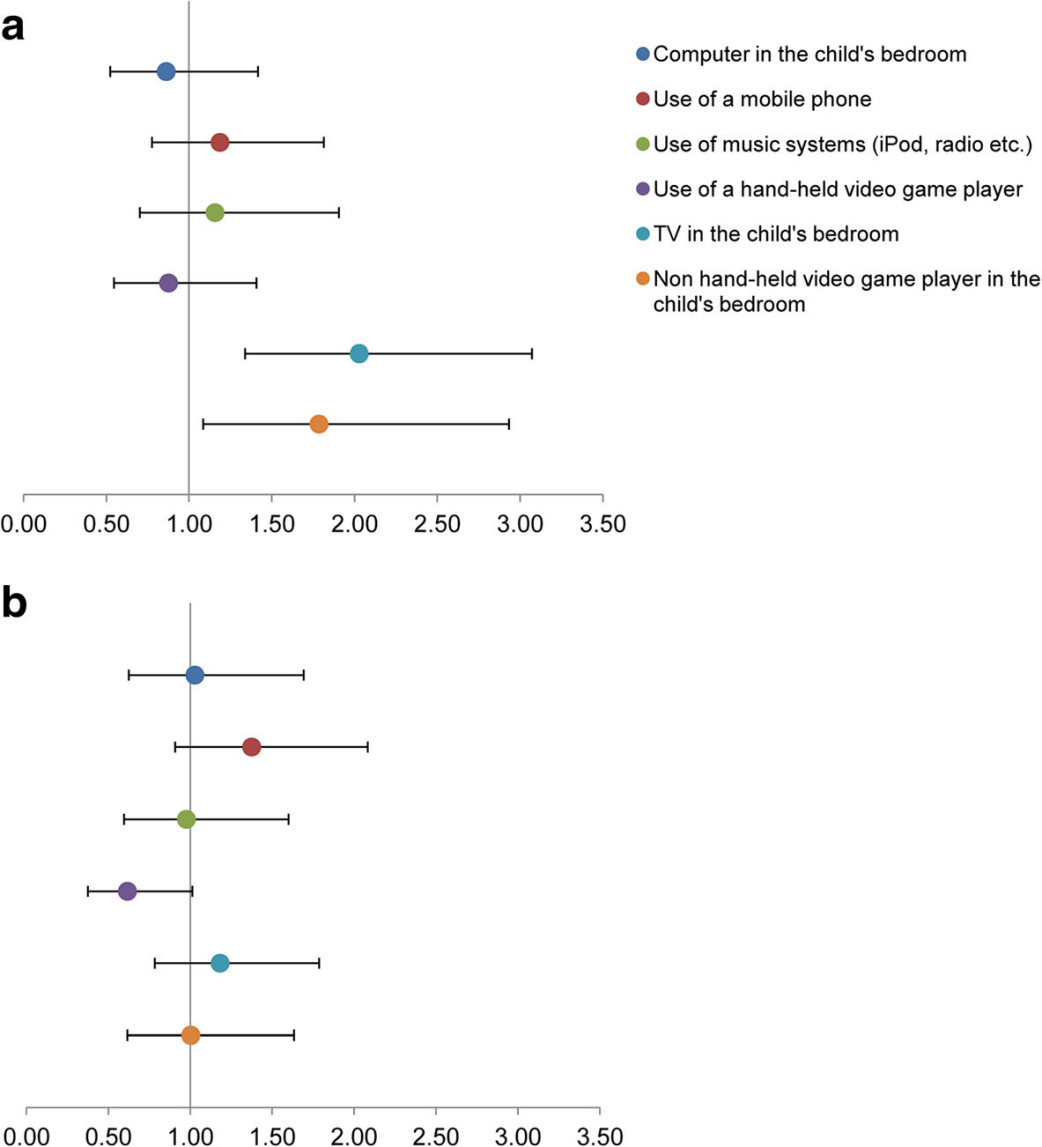
Odds associated with spending (**a**) > 1 h outside after school; (**b**) > 2 h outdoors on a weekend, according to whether children have access to specific electronic media devices or not (reference category = no access), adjusting for age, sex and socioeconomic status (highest parental education level), with schools treated as random effects

The odds of spending more time outdoors, according to whether participants had access to specific media devices or not, are shown in Fig. 1. Only two relationships were statistically significant: in comparison to those who did not have a TV in their bedroom, children who did were 2.03 (1.34–3.07; *p* = 0.001) times more likely to spend > 1 h outside after school, and children with a non-handheld video game player (e.g., PlayStation, Xbox etc.) in their bedroom were 1.79 (1.09–2.93; *p* = 0.022) times more likely to spend this long outdoors after school. No significant associations were found for weekend outdoor time, although the association for use of a handheld video game player approached significance; children with such a device were less likely to spend > 2 h outdoors on weekend days than those without one (OR = 0.62, 0.38–1.01; *p* = 0.056).

## Discussion

The aim of this study was to extend previous research on children’s physical activity, by exploring potential correlates of time spent outdoors, a consistent correlate of children’s physical activity, [3] during specific periods when children have more free choice over their behaviour. The results provide new insight into the relationships between parental safety concerns, screen-based behaviours, and time spent outdoors after school and on weekends. Group differences were also found, as well as interactions by sex and SES.

Boys and low SES children were more likely to spend > 1 h outside after school than girls and high SES participants, respectively. These relationships did not hold for time spent outside on weekends, but older children were more likely to spend > 2 h outdoors during this time, although this relationship was only significant among girls and high SES participants. These results are interesting as they align with previous research on independent mobility showing that boys, [16, 28, 40–43] children of a low SES background, [42–45] and older children, [16, 42, 43, 45] tend to be given greater freedom to roam. As such, independent mobility may be an underlying mechanism that was at play in our study, but more research is needed to confirm this, since independent mobility was not assessed here.

In terms of research on time outdoors specifically, our results concur with past work showing that boys tend to spend more time outdoors than girls [6, 23, 28, 31]. As for socioeconomic differences, a negative relationship between parental education and time outdoors was reported among 3–4 year olds in the United States (US), [24] and among children aged 7–12 years in the Netherlands, but no association was found for 4–6 year olds within the same study [22]. No significant difference was reported in another US study on pre-schoolers aged 2–5 years, [23] thus conflicting results have previously been reported. Age differences, other sample characteristics, and variations in the way that time spent outdoors is assessed, could explain these discrepancies. Research on UK children, exploring socioeconomic disparities in time spent outdoors is needed to support our findings. Future work would also do well to explore the reasons for any such divide between socioeconomic groups because they could point to differences in social norms, availability of technologies, or the built environment, which would help to improve future intervention design aimed at particular groups. For example, parents from different socioeconomic backgrounds may have different attitudes towards allowing their children to play outside, and this in turn may be influenced by whether there are spaces considered to be safe for outdoor play, such as a garden or a quiet cul-de-sac.

According to previous research, a weak negative relationship between sedentary behaviour and physical activity exists, [19, 46] yet little is known about the relationship between screen-based behaviours and outdoor time specifically. Our findings suggest that the time spent playing on a computer is more important than the time watching TV, because a negative association was observed for computer use and outdoor time after school, whereas no overall effect for TV viewing was found. However, there were differences between the ‘extreme’ groups in that participants who watched a low amount of TV (< 2 h/day) on school days were less likely to spend > 1 h outside after school than those watching a high amount of TV (≥ 3 h/day).

In addition, access to a TV or non-handheld video game player in the bedroom was associated with higher odds of time outdoors after school in comparison to children without these. Similar findings were reported in a study of 7 year old children; the presence of a TV in the bedroom was positively associated with physical activity [47]. The authors speculated that this may have been a marker of SES, as they also found low SES participants to be more active than their high SES counterparts [47]. Yet, no significant interaction by SES was found for any of the sedentary pursuits explored in this study, so other factors could be at play. For example, children with access to very few electronic media devices or who watch very little TV, may live in households where screen time is more carefully monitored and/or highly prohibited. It is therefore possible that other rules and restrictions may be enforced, such as not being allowed to play outdoors unsupervised. Indeed, previous research has shown that greater restrictions on sedentary behaviours are negatively associated with physical activity [48, 49]. Further research is needed to test whether this mechanism is feasible or whether other reasons, such as reverse causality, [48] for these counter-intuitive findings are at play.

Nevertheless, our findings raise questions about the potential efficacy of previous strategies, such as the removal of devices from the bedroom or TV limiting devices, [50, 51] that are proposed to increase children’s physical activity levels. However, such proposals are based on results showing that greater access to media devices in the child’s bedroom is negatively associated with children’s physical activity and positively with sedentary time, [44, 49] which contrasts with our findings. Such differences may simply be due to the fact that we assessed outdoor time specifically, as opposed to overall physical activity, and it is possible that the children with access to more electronic devices in our study, may have spent more time outdoors inactive (e.g., engaging in social sedentary behaviours). Thus they may not necessarily be more active despite spending more time outside. Alternatively, there may be variations in the way that electronic media devices are reported (i.e., via child versus parent reports), [52] and devices in the child’s bedroom may have contrasting effects to those that are portable in nature. As children have access to a number of sedentary screen-based devices, [38] new research exploring the impact of time spent in other screen-based pursuits (e.g., tablet computers and smart phones) is needed as it will be important to know whether they add to, or replace, the use of existing devices already present in the home.

In terms of parental safety perceptions, a significant negative association was evident for traffic-related concerns and time outdoors after school among boys. This contrasts with the results of another UK study, whereby a negative relationship between traffic safety concerns and time spent outside was apparent among girls only [53]. It is unknown why such a relationship was not observed for girls in this study, but it could be because fewer girls spent several hours outside during this time than boys. Furthermore, in contrast to our approach, in their study the authors examined outdoor time across the whole week as well as children’s perceptions of safety as opposed to parental perceptions [53]. Again, such discrepancy in design may explain differences when compared to the present work. Nonetheless, it may be that road safety strategies targeting boys’ safe play or active transport around the neighbourhood may be needed among those who have parents that restrict their outdoor time. Though, safety measures should be enforced such that they do not compromise on children’s ability to partake in unsupervised outdoor play [8, 54].

As for crime-related concerns, the results were less intuitive because higher concerns of this kind were associated with increased odds of time outside after school in this sample. It is possible that parents with more concerns experience these because their children spend more time outdoors, thus they may be more aware of potential dangers [55]. Equally, it may be that such parents do not restrict their child’s behaviour despite feeling concerned. They may therefore have effective coping strategies; if so qualitative studies which look to explore these would be useful as they may provide a means for overcoming concerns among parents who do restrict their child’s outdoor time. On the other hand, it may be that these children spend more time outdoors in protest to any restrictions placed upon them, [56] though this seems unlikely given the age group being studied. A more plausible explanation is that parents who report greater concerns may simply supervise their children’s outdoor play or active travel [56]. This emphasises the importance of taking the whole context into account, by specifying where and with whom such behaviour takes place, as well as the need for longitudinal research given that the direction of this relationship is unknown because of the cross-sectional study design.

### Limitations

This study is also limited by the use of self-reported measures to assess all variables included in the analysis and the questions used to measure time spent outdoors in particular were developed by the ISCOLE team, thus they have not been assessed for validity or reliability [27]. It has been proposed that mixed methods designs, including both objective and subjective measures of outdoor play, and a standardized measurement tool should be employed in future research [57]. Furthermore, our measure of time spent outdoors does not provide any information on the actual behaviours undertaken outdoors (e.g., walking, skating, playing, sitting etc.), nor did we capture information on where such behaviour took place (e.g., on the streets, in the school playground, in the garden etc.). Future work should take these factors into consideration as they may point to possible explanations for some of the group differences and counterintuitive findings reported in this study. However, a strength of this work is the inclusion of time-specific data for both screen-based pursuits and time spent outdoors, which can help to improve the predictive capacity of the relationships being tested [58].

It is possible that other factors not studied here may play an important role (e.g., access to facilities, parent support, seasonality etc.) that should also be explored in future. Further, the majority of the sample classified themselves as White British (87.9%), and data were not collected on children living in rural areas. Consequently, the findings of this study may not generalise to other populations and important differences between ethnic groups or between urban and rural settings could not be assessed. Parental perceptions of the neighbourhood environment are more likely to be influential in this age group, though future studies should explore the impact of both children’s and parent’s perceptions, as they may have independent and/or interacting effects [12, 53].

## Conclusions

In conclusion, our results show that certain groups of children (i.e., girls, high SES children, younger age groups, and those who play on a computer for long periods of time) are at greater risk of spending their free time indoors, which could have important health implications with regards to their development and physical activity levels. Thus, interventions designed to promote physical activity among such groups may benefit from increasing their time outdoors during discretionary periods. Some counterintuitive findings were also reported in terms of the electronic media environment and crime-related safety concerns because positive associations with these and time outdoors after school were found. Further research, including longitudinal studies, is needed to test some of the proposed mechanisms that may be at play in order to explain these results.

## Abbreviations

GCSE: General Certificate for Secondary Education
ISCOLE: International Study of Childhood Obesity, Lifestyle and the Environment
SES: Socioeconomic status
TV: Television
UK: United Kingdom
US: United States
